# Optimization of a dedicated protocol using a small-voxel PSF reconstruction for head-and-neck ^18^FDG PET/CT imaging in differentiated thyroid cancer

**DOI:** 10.1186/s13550-018-0461-x

**Published:** 2018-12-03

**Authors:** Renaud Ciappuccini, Cédric Desmonts, Idlir Licaj, Cécile Blanc-Fournier, Stéphane Bardet, Nicolas Aide

**Affiliations:** 1 Department of Nuclear Medicine and Thyroid Unit, François Baclesse Cancer Centre, 3 Avenue Général Harris, 14000 Caen, France; 2 INSERM 1086 ANTICIPE, Normandie University, Caen, France; 3 Department of Nuclear Medicine, University Hospital, Caen, France; 4 Department of Clinical Research, François Baclesse Cancer Centre, Caen, France; 5 Department of Pathology, François Baclesse Cancer Centre, Caen, France

**Keywords:** ^18^FDG PET/CT, List-mode acquisition, Point spread function, Head and neck imaging, Thyroid cancer

## Abstract

**Background:**

^18^FDG PET/CT is crucial before neck surgery for nodal recurrence localization in iodine-refractory differentiated or poorly differentiated thyroid cancer (DTC/PDTC). A dedicated head-and-neck (HN) acquisition performed with a thin matrix and point-spread-function (PSF) modelling in addition to the whole-body PET study has been shown to improve the detection of small cancer deposits. Different protocols have been reported with various acquisition times of HN PET/CT. We aimed to compare two reconstruction algorithms for disease detection and to determine the optimal acquisition time per bed position using the Siemens Biograph6 with extended field-of-view.

**Methods:**

Twenty-one consecutive and unselected patients with DTC/PDTC underwent HN PET/CT acquisition using list-mode. PET data were reconstructed, mimicking five different acquisition times per bed position from 2 to 10 min. Each PET data set was reconstructed using 3D-ordered subset expectation maximisation (3D-OSEM) or iterative reconstruction with PSF modelling with no post filtering (PSF_allpass_). These reconstructions resulted in 210 anonymized datasets that were randomly reviewed to assess ^18^FDG uptake in cervical lymph nodes or in the thyroid bed using a 5-point scale. Noise level, maximal standard uptake values (SUVmax), tumour/background ratios (TBRs) and dimensions of the corresponding lesion on the CT scan were recorded. In surgical patients, the largest tumoral size of each lymph node metastasis was measured by a pathologist.

**Results:**

The 120 HN PET studies of the 12 patients with at least 1 ^18^FDG focus scored malignant formed the study group. Noise level significantly decreased between 2 and 4 min for both 3D-OSEM and PSF_allpass_ reconstructions (*p* < 0.01). TBRs were similar for all the acquisition times for both 3D-OSEM and PSF_allpass_ reconstructions (*p* = 0.25 and 0.44, respectively). The detection rate of malignant foci significantly improved from 2 to 10 min for PSF_allpass_ reconstruction (20/26 to 26/26; *p* = 0.01) but not for 3D-OSEM (15/26 to 19/26; *p* = 0.26). For each of the five acquisition times, PSF_allpass_ detected more malignant foci than 3D-OSEM (*p* < 0.01). In the seven surgical patients, PSF_allpass_ evidenced smaller malignant lymph nodes than 3D-OSEM at 8 and 10 min. At 10 min, the mean size of the lymph node metastases neither detected with PSF_allpass_ nor 3D-OSEM was 3 ± 0.6 mm vs 5.8 ± 1.1 mm for those detected with PSF_allpass_ only and 10.9 ± 3.3 for those detected with both reconstructions (*p* < 0.001).

**Conclusions:**

PSF_allpass_ HN PET improves lesion detectability as compared with 3D-OSEM HN PET. PSF_allpass_ with an acquisition time between 8 and 10 min provides the best performance for tumour detection.

## Background

^18^FDG PET/CT is crucial before neck surgery for nodal recurrence detection in radioiodine-refractory differentiated or poorly differentiated thyroid cancer (DTC/PDTC), as the surgeon’s plan is mainly guided by PET/CT and neck US data. In this setting, a dedicated head-and-neck PET/CT acquisition (HN PET) performed with a thin matrix and point-spread-function (PSF) modelling in addition to the whole-body (WB) PET study has been shown to improve the detection of small cancer deposits [1]. Various HN PET/CT protocols have been reported for head-and-neck malignancies including thyroid cancers, with different algorithm reconstructions (mainly without PSF) and with different acquisition times ranging from 6 to 15 min [1–5]. In this context, to manage patients’ schedules of a busy PET unit, it would be helpful to optimise the acquisition time of such a complementary HN PET acquisition for routine practice. The goal would be to determine the acquisition time that allows high sensitivity for disease detection, without uselessly increasing the whole PET acquisition time. Furthermore, PSF and ordered subset expectation maximisation (OSEM) reconstructions have not been compared for head-and-neck (HN) imaging with various acquisition times. We aimed to compare these two reconstruction algorithms for the detection of thyroid cancer recurrence in the neck and to determine the optimal acquisition time per bed position using the Siemens Biograph6 with extended field-of-view.

## Methods

### Patient selection

Twenty-one consecutive and unselected patients with DTC/PDTC who underwent an ^18^FDG PET/CT scan with a complementary dedicated HN PET/CT acquisition between June 2015 and November 2017 in our department were reviewed. All had a negative post-therapeutic ^131^I WB scan.

### PET/CT

PET/CT acquisitions were performed using a PET/CT scanner (Biograph TrueV, Siemens Medical Solutions) with a six-slice spiral CT component and an extended field-of-view of 21.6 cm. Patients were asked to fast for ≥ 6 h before ^18^FDG injection (4.0 ± 0.2 MBq per kg). Blood glucose level was 6.0 ± 1.7 mmol/l. WB PET/CT images were performed 58 ± 3 min post injection from mid-thigh to the base of the skull with an acquisition time per bed position of 2 min and 40 s in patients of low and average weight (i.e. body mass index [BMI] < 25 kg/m^2^) or 3 min and 40 s in overweight patients (i.e. BMI ≥ 25 kg/m^2^). HN PET was performed either immediately after the WB acquisition and patient repositioning (3 ± 2 min between WB and HN PET, 14 patients) or after the WB acquisition of the next-scheduled patient (18 ± 5 min between WB and HN PET, 7 patients). HN PET/CT data sets were acquired in list-mode (LM) using a single-bed position of 10 min from the base of the skull to the superior mediastinum. All patients were repositioned with arms along the chest. A low-dose CT scan was acquired prior to HN PET/CT with the same longitudinal field of view. CT parameters were set to 100 mAs and 130 kV, slice thickness of 2.5 mm and pitch 1.

### Image reconstruction

PET raw data were reconstructed with the PSF reconstruction algorithm (HD; TrueX, Siemens Medical Solutions; 3 iterations and 21 subsets) without filtering (PSF_allpass_) [6] and with the 3D-OSEM reconstruction algorithm (4 iterations and 8 subsets). Scatter and attenuation corrections were carried out. For all HN PET reconstructions, matrix size was 256 × 256 (vs. 168 × 168 for WB acquisition), resulting in a 2.67 × 2.67 × 2.67 mm voxel size (small-voxels). For each PSF_allpass_ or 3D-OSEM reconstruction, five datasets were reconstructed from this LM acquisition, from 2 to 10 min with a 2-min increment. Overall, ten HN PET data sets were obtained per patient.

### ^18^FDG-PET/CT scan interpretation

All images were blindly reviewed after randomisation by an experienced nuclear medicine physician on a digital workstation (eSoft/TrueD workstation, Siemens Medical Solutions). The randomisation was performed as follows: all data sets of 2 min were first randomly pooled together, and then those of 4, 6, 8 and 10 min. Overall, 210 anonymized PET data sets were reviewed during separate reading sessions (8 weeks apart).

The reader reported each ^18^FDG focus on HN PET images and graded its uptake on a scale of 1–5 (1, definitely benign; 2, probably benign; 3, indeterminate; 4, probably malignant; and 5, definitely malignant). Maximum standardised uptake value (SUVmax) was measured according to EANM guidelines [7]. Mean standardised uptake value (SUVmean) of the vascular background was measured in the large neck vessels (carotid artery and internal jugular vein). The noise was calculated as follows: $\frac{\mathrm{standard}\ \mathrm{derivation}\ \mathrm{of}\ \mathrm{SUV}\ \mathrm{in}\ \mathrm{the}\ \mathrm{vascular}\ \mathrm{background}\ }{\mathrm{SUVmean}\ \mathrm{in}\ \mathrm{the}\ \mathrm{vascular}\ \mathrm{background}}\times 100$. The tumour/background ratio (TBR) of an ^18^FDG focus was computed as follows: $\frac{\mathrm{SUVmax}\ \mathrm{in}\ \mathrm{the}\ \mathrm{lymph}\ \mathrm{node}\ \mathrm{or}\ \mathrm{the}\ \mathrm{local}\ \mathrm{tumour}\ }{\mathrm{SUVmean}\ \mathrm{in}\ \mathrm{the}\ \mathrm{vascular}\ \mathrm{background}.}$

Lymph nodes or thyroid bed lesions corresponding to ^18^FDG foci identified on co-registered CT images were also reported.

At the patient level, the HN PET/CT study was scored either negative (^18^FDG foci all scored 1 or 2), indeterminate (no ^18^FDG focus scored higher than 3), or positive (≥ 1^18^FDG focus scored 4 or 5).

Surgery and follow-up were the reference standard for ^18^FDG foci at head-and-neck level.

Pathology was the gold standard in surgical patients. The largest tumoral size of each lymph node metastasis was estimated by one pathologist.

### Statistical analysis

Quantitative data are expressed in mean ± standard deviation (SD), or median (min-max). The nonparametric Mann-Whitney *U* test for paired samples was used to compare noise levels, TBRs, and SUVmax at different time points. For all tests, a two-tailed *p* value < 0.05 was considered statistically significant. Graphs and statistics were performed using Prism (GraphPad software, La Jolla, CA) and Vassar University clinical research calculators (http://vassarstats.net/).

## Results

### Patients’ characteristics

Of the 21 DTC/PDTC patients, 16 had neck US abnormalities and/or biochemical disease (elevated serum thyroglobulin [Tg] levels or rising anti-Tg antibodies [TgAb] levels), three had metastatic disease under surveillance and two were at high-risk [8] before first radioiodine administration.

Twelve out of 21 patients had at least 1 ^18^FDG uptake suggestive of a tumour (i.e. score ≥ 4) on HN PET. All the 26 ^18^FDG findings of score ≥ 4 in these 12 patients were confirmed as truly malignant after surgery (*n* = 7) or follow-up on PET/CT (*n* = 5).

Of the nine patients with a negative PET/CT, five had a persistent biological disease without persistent/recurrent disease (PRD) during follow-up, two had ^18^FDG-negative lymph node involvement with positive US (pathological confirmation after surgery), one had a ^18^FDG true-negative study with false-positive lymph node involvement on US (confirmed as benign after surgery) and one had a subsequent ^18^FDG-positive PET/CT study after biological disease progression during follow-up.

### Noise level

**Fig. 1:**
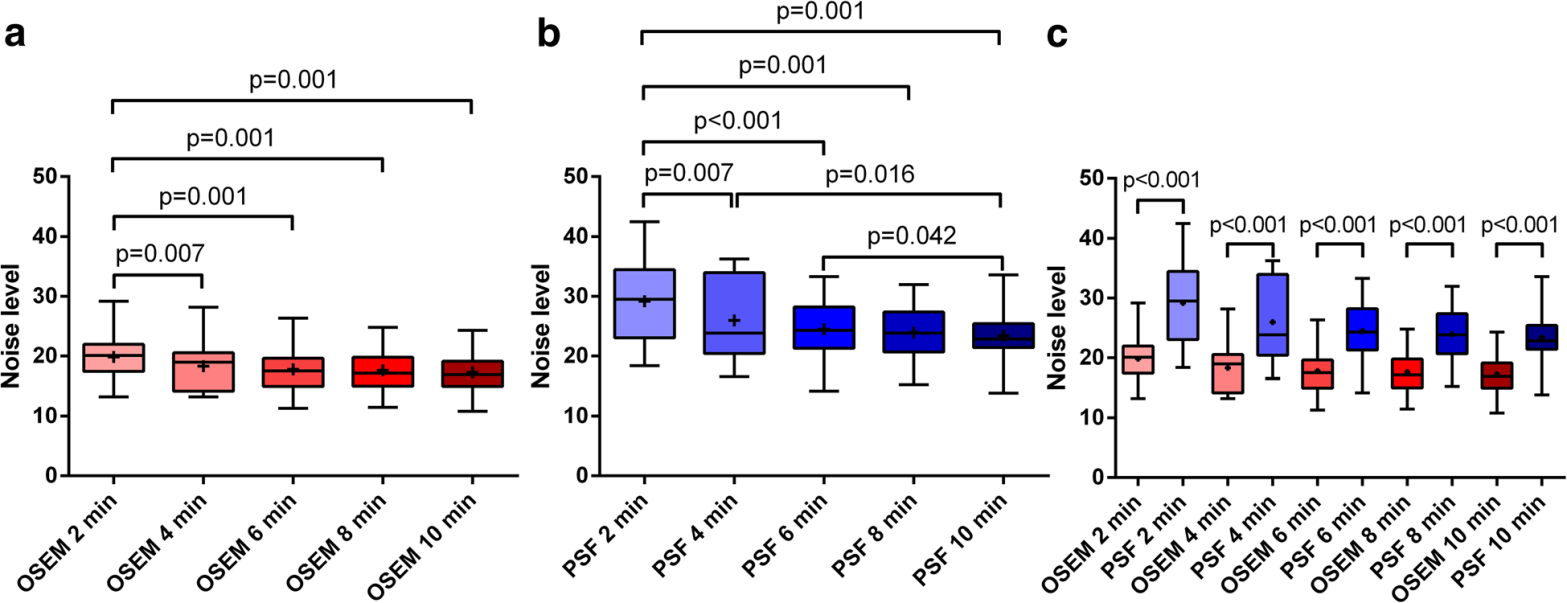
Noise level measured in the vascular background in 3D-OSEM-reconstructed (**a**) and PSF_allpass_-reconstructed (**b**) head-and-neck PET data sets for each of the five acquisition times per bed position from 2 to 10 min with a 2-min increment. **c** Comparison of the noise level between 3D-OSEM and PSF_allpass_-reconstructed head-and-neck PET data sets

For both 3D-OSEM and PSF_allpass_ reconstructions, the noise significantly decreased between 2 and 4 min (*p* = 0.007). Also, the noise significantly decreased for PSF_allpass_ reconstruction between 2 and 10 min (*p* = 0.016), and between 4 and 10 min (*p* = 0.042) (Fig. 1a, b).

The noise was significantly higher with PSF_allpass_ than 3D-OSEM reconstruction for each of the five acquisition times per bed position from 2 to 10 min (Fig. 1c).

### Tumour/background ratios

**Fig. 2:**
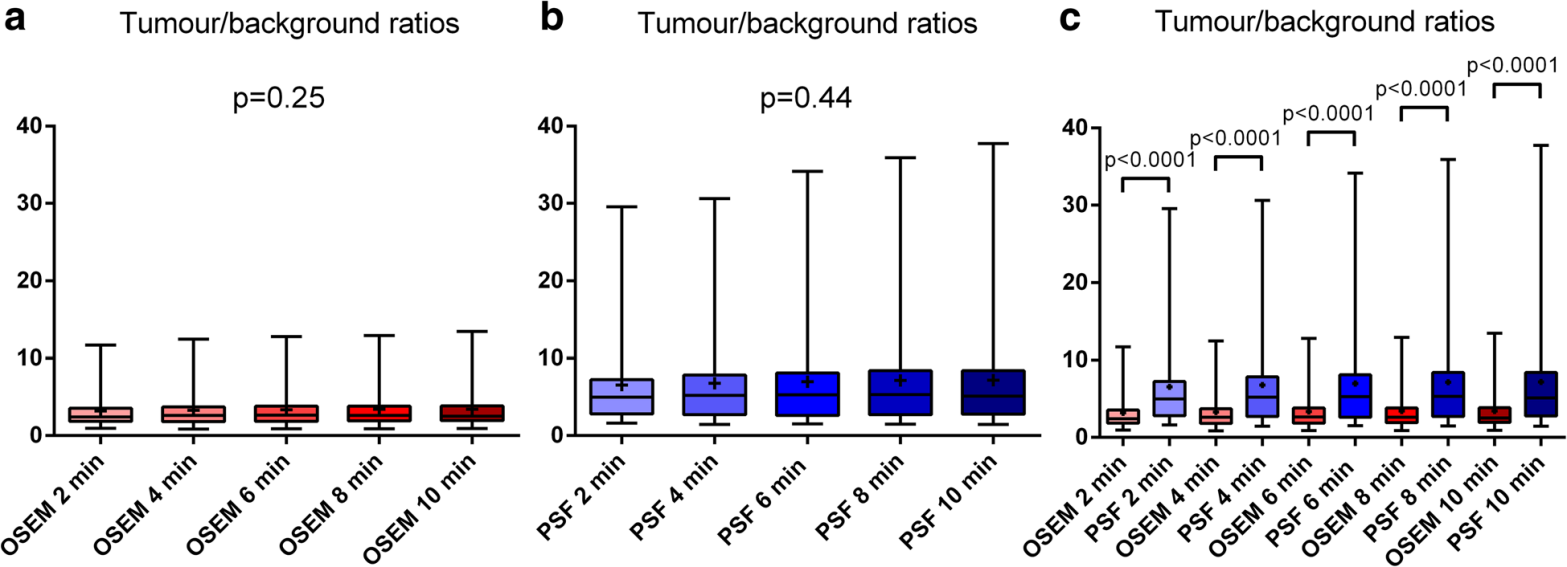
Comparison of the tumour/background ratios (TBRs) in 3D-OSEM-reconstructed (**a**) and PSF_allpass_-reconstructed (**b**) head-and-neck (HN) PET data sets for each of the five acquisition times per bed position from 2 to 10 min with a 2-min increment. **c** Comparison of the TBRs between 3D-OSEM and PSF_allpass_-reconstructed HN PET data sets

For 3D-OSEM reconstruction, TBRs were similar for all acquisition times per bed position (*p* = 0.25). The same results were observed for PSF_allpass_ reconstruction (*p* = 0.44) (Fig. 2a, b). TBRs were higher with PSF_allpass_ than with 3D-OSEM reconstruction for each of the five acquisition times per bed position (Fig. 2c).

**Fig. 3:**
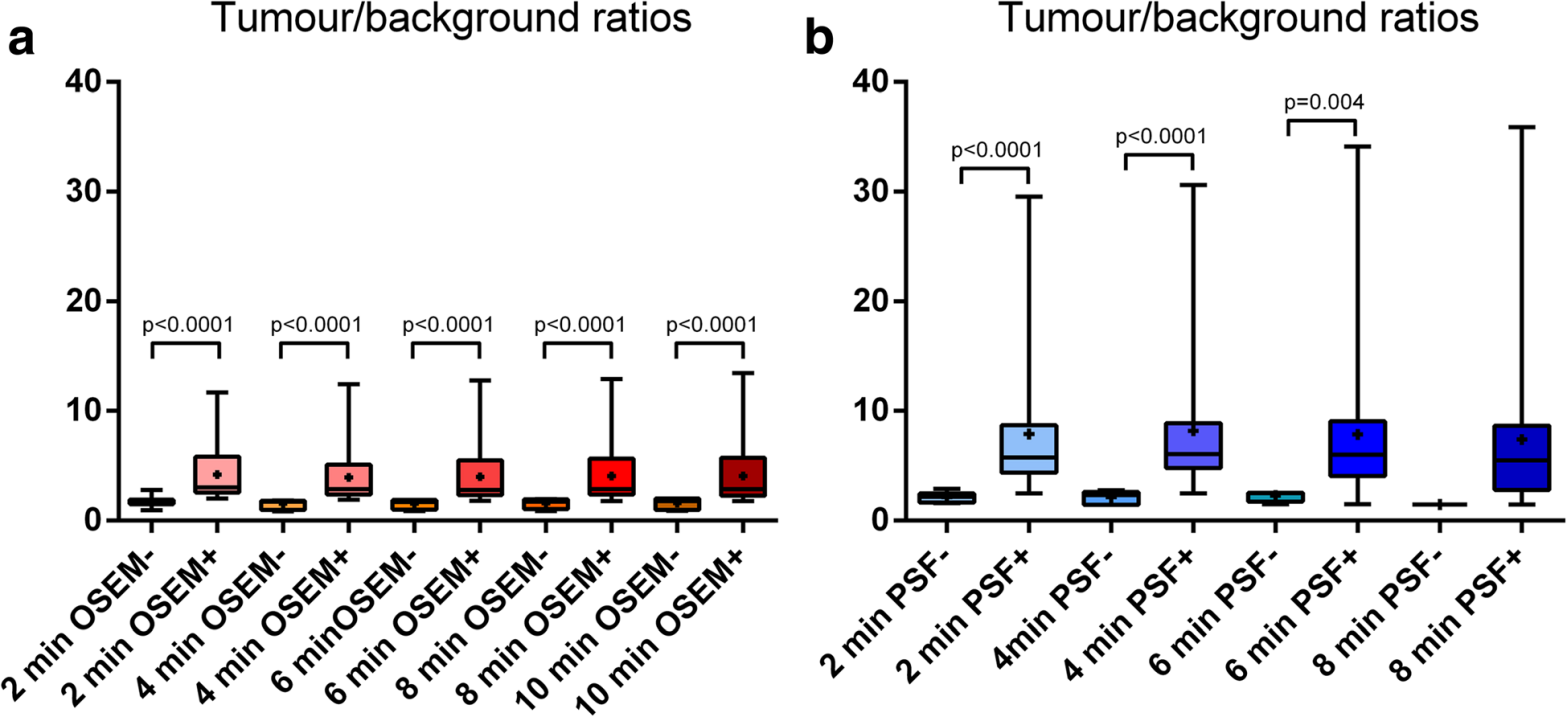
Comparison of the tumour/background ratios (TBRs) from 2 to 10 min between ^18^FDG-foci evidenced (+) or not (−) in 3D-OSEM-reconstructed (**a**) and PSF_allpass_-reconstructed (**b**) head-and-neck PET data sets. The comparison was not performed at 8 min for PSF_allpass_-reconstructed images because of the number of values in the PSF− group (*n* = 2)

For both 3D-OSEM and PSF_allpass_ reconstructions, TBRs were significantly higher in detectable lesions than in undetectable lesions for each of the five acquisition times per bed position (Fig. 3).

**Fig. 4:**
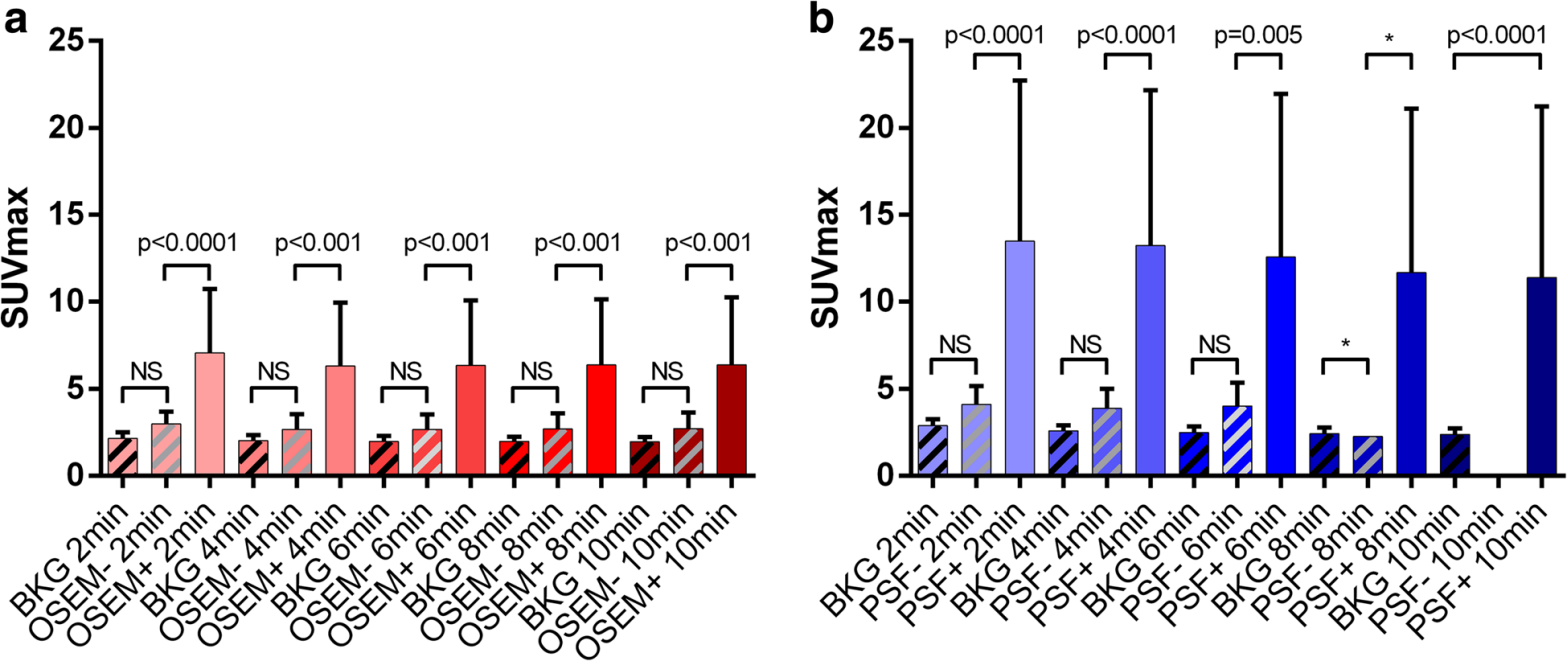
Comparison of maximal standard uptake values (SUVmax) (mean, SD) between vascular background (BKG) and ^18^FDG-foci suggestive of tumour detected or not on 3D-OSEM (**a**) and PSF_allpass_ (**b**) reconstructed PET images. PSF− are lesions not detected by PSF_allpass_, OSEM− are those not detected by 3D-OSEM. Conversely, PSF+ are lesions detected by PSF_allpass_ and OSEM+ are those detected by 3D-OSEM. NS: non-significant; * the number of ^18^FDG-foci in the PSF− group (*n* = 2) does not enable statistical analysis

For each acquisition time and for each reconstruction, the SUVmax of the lesions not detected were not statistically different from the SUVmax measured in the vascular background (Fig. 4).

### Lesion detectability

**Fig. 5:**
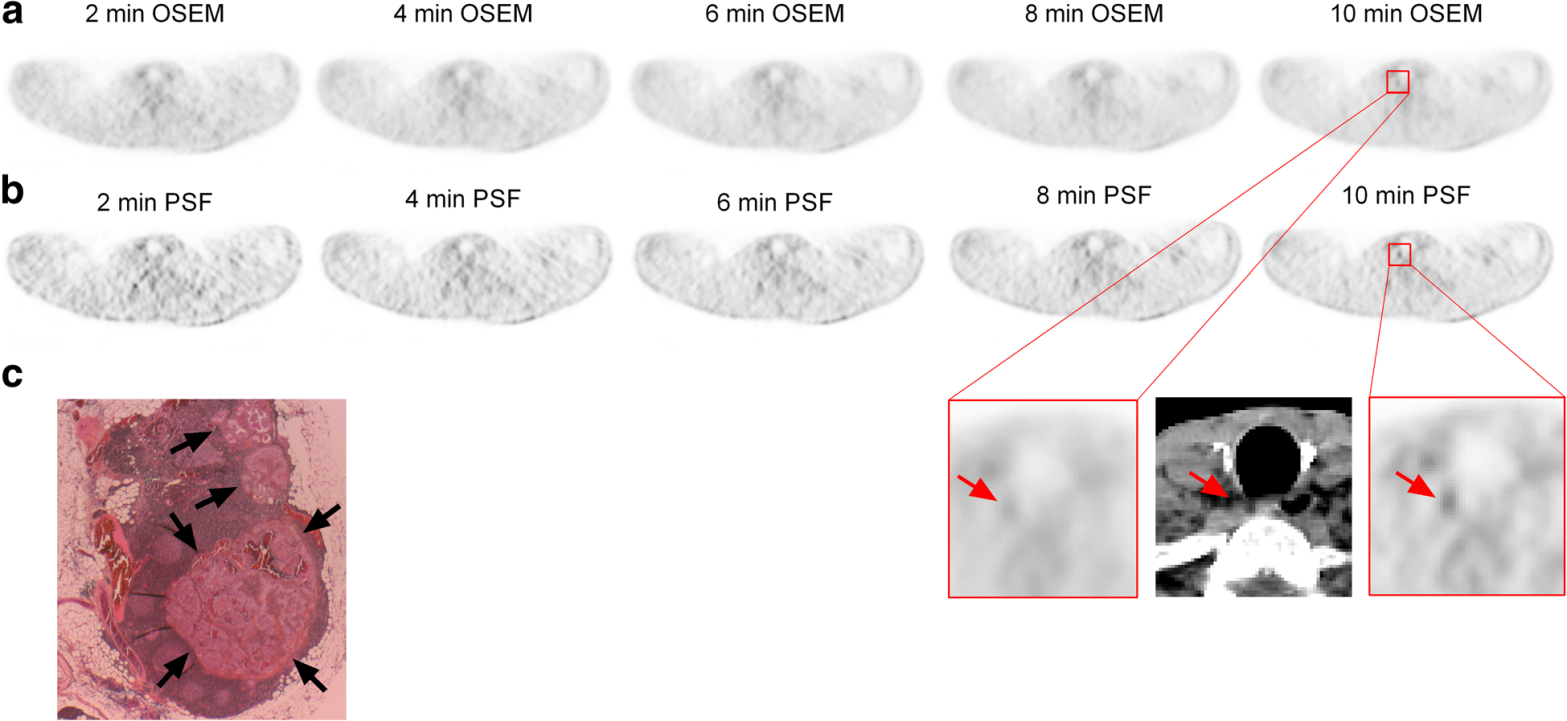
A 40-year-old male patient with a 20-mm papillary DTC was referred for ^18^FDG PET/CT in November 2015. He had previously undergone total thyroidectomy in 2001 and remission was observed during yearly follow-up until 2014. In 2015, the serum Tg was still < 0.04 ng/ml but serum TgAb appeared. Head-and-neck PET was performed. Axial PET on the same level was shown with 3D-OSEM (**a**) and PSF_allpass_ (**b**) reconstructions. No abnormal ^18^FDG focus was reported on 3D-OSEM-reconstructed HN PET (at 10 min: SUVmax = 1.44, TBR = 0.99). On the PSF_allpass_-reconstructed HN PET data sets, a faint focal ^18^FDG uptake in the right-sided central compartment was scored as probably malignant (score 4) at 10 min (SUVmax = 2.26, TBR = 1.46), corresponding to a 5 × 3 mm lymph node on CT scan (red arrows). **c** Pathology confirmed that this abnormal focus was truly malignant. The size of the tumour deposit in the right central lymph node was 5 mm (HES staining, × 2.5; black arrows)

**Fig. 6:**
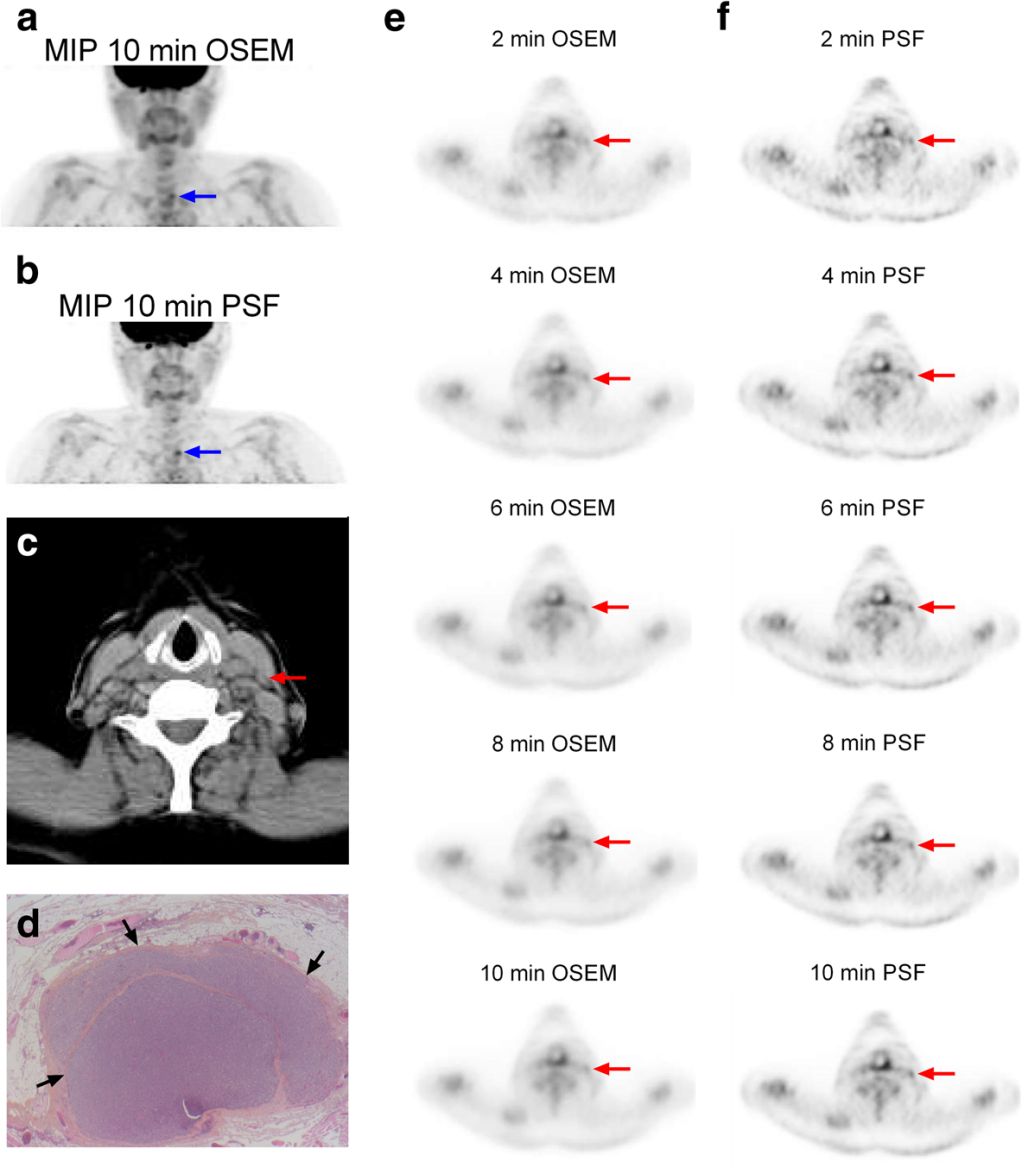
A 62-year-old female patient with a 70-mm PDTC (pT3 Nx Mx) was referred for ^18^FDG PET/CT in November 2017 to explore a detectable serum Tg level under levothyroxine (11 ng/ml, without serum TgAb) 2 years after initial ^131^I treatment. Maximum intensity projection images (MIP) showed lymph node involvement of the left central compartment on both 3D-OSEM-reconstructed HN PET (**a**, blue arrow) and PSF_allpass_-reconstructed HN PET (**b**, blue arrow). No abnormal ^18^FDG focus (score ≤ 3) was reported on 3D-OSEM-reconstructed HN PET in the left lateral compartment (at 10 min: SUVmax = 1.67, TBR = 1.19) (**e**). On the PSF_allpass_-reconstructed HN PET data sets (**f**), a small focal ^18^FDG uptake in the left lateral compartment was scored as probably malignant (score 4) at 4–10 min (at 10 min: SUVmax = 2.43, TBR = 1.74), corresponding to a 6 × 4 mm lymph node on CT scan (**c**, red arrow). Left central and left lateral dissection was performed and confirmed lymph node involvement in both compartments. **d** The left lateral lymph node was massively invaded (black arrows). The size of the tumour deposit was 5 mm (HES staining, × 2.5)

For each acquisition time from 2 to 10 min, 3D-OSEM reconstruction detected 15, 19, 19, 19 and 19 lesions, respectively (*p* = 0.26), while PSF_allpass_ reconstruction detected 20, 20, 22, 25 and 26 lesions, respectively (*p* = 0.01). Figures 5 and 6 present examples of mismatches between 3D-OSEM and PSF_allpass_ HN PET data sets reading.

When comparing both reconstructions at each of the five acquisition times per bed position from 2 to 10 min, PSF_allpass_ performed better than 3D-OSEM for lesion detection (*p* < 0.01, *p* < 0.0001, *p* < 0.01, *p* = 0.02 and *p* < 0.01, respectively).

**Fig. 7:**
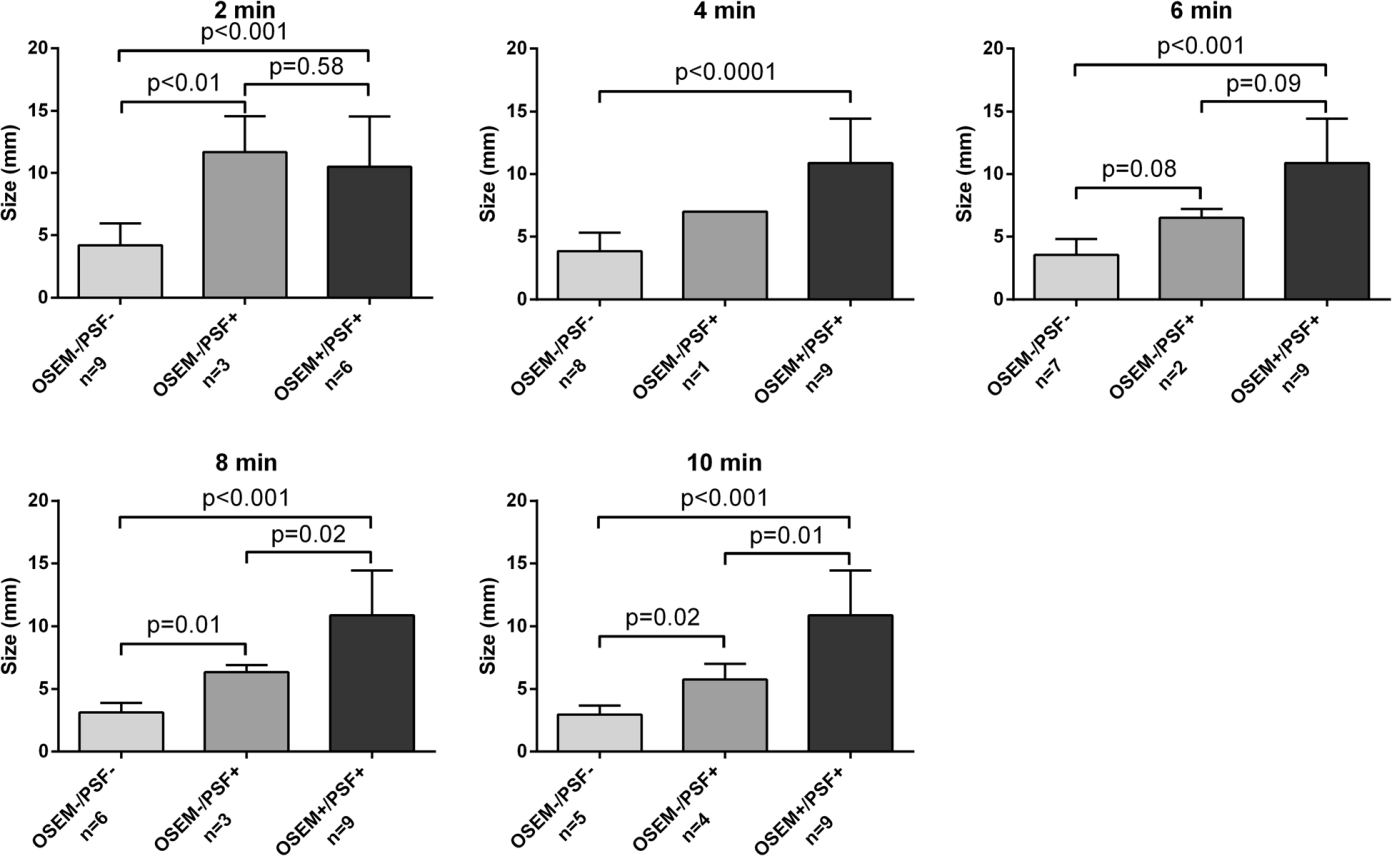
Comparison of the size (mean, SD) of the lymph node metastases detected or not on the ^18^FDG PET/CT scans at each acquisition duration time from 2 to 10 min depending on the reconstruction modality in surgical patients. OSEM-/PSF- are lesions not detected by either reconstruction, OSEM-/PSF+ are those detected only by PSF_allpass_ reconstruction, and OSEM+/PSF+ are those detected by both reconstructions

In the seven surgical patients, PSF_allpass_ detected smaller malignant lymph nodes than 3D-OSEM at 8 and 10 min (Fig. 7). At 10 min, the mean size of the lymph node metastases neither detected with PSF_allpass_ nor 3D-OSEM was 3 ± 0.6 mm vs 5.8 ± 1.1 mm for those detected with PSF_allpass_ only and 10.9 ± 3.3 mm for those detected with both reconstructions (*p* < 0.001).

## Discussion

To date, several PET/CT studies have explored recurrent disease in the neck for thyroid malignancies with various protocols using WB or HN PET, different acquisition times, reconstruction algorithms or delayed images. The results of these studies are difficult to compare to each other. To our knowledge, no study has previously determined the optimal acquisition time using PSF reconstruction. We observed that 4 min was the minimal acquisition time for both PSF_allpass_ and 3D-OSEM HN PET to overcome the noise level. We further showed that an increase of the PSF_allpass_ acquisition time to either 8 or 10 min provided the best performance for lymph node or local recurrence diagnosis in DTC/PDTC.

This study is the first to compare PSF modelling to conventional OSEM reconstruction with different acquisition times at the HN level. The protocol was designed for routine practical application. Indeed, adding a 10-min HN PET acquisition after the WB PET can be easily performed even in busy PET units. We have recently demonstrated that an HN PET acquisition offers a better diagnostic value than WB PET alone because of the following advantages: thinner matrix, longer acquisition time and reduced motion artefacts [1]. However, the previous studies for HN squamous cell cancers and thyroid malignancies reported a wide range of acquisition times for HN PET: 6 min [4], 8 min [1], 10 min [2], 12 min [5] or 15 min [3]. Thus, it seemed crucial to determine the time needed for an HN PET acquisition. In our study protocol, the longest acquisition for HN PET was 10 min because we felt that a dedicated acquisition over 10 min was not suitable for routine practice to respect the patients’ schedule and to fulfil good practice and EARL guidelines [7, 9]. Furthermore, our results can apply to both PET devices equipped or not with PSF reconstruction. PSF has been shown to increase the detection of small cancer lesions such as lymph node metastases in breast [10] and lung cancers [11]. Our data confirmed this finding for PRD in DTC/PDTC patients at the HN level.

We showed that although 3D-OSEM 4-min performances were close to those of PSF_allpass_ at 4 min, a two-fold increase of the acquisition time with PSF_allpass_ (i.e. 8 min) considerably increased ^18^FDG foci detection. Indeed, ^18^FDG foci detection continued to increase for PSF_allpass_ after 4 min, whereas there was no significant added value in increasing the acquisition time for 3D-OSEM after 4 min. Detecting all malignant ^18^FDG foci is of importance for the surgeon because the treatment plan (i.e. the number and the localization of the neck compartments to dissect) is affected by PET data. The limited number of surgical patients in the present study did not enable an investigation in which proportion the treatment plan was modified.

Several factors can account for better sensitivity with PSF_allpass_ than 3D-OSEM with time. First, the noise level significantly decreased between 2 and 4 min for both 3D-OSEM and PSF_allpass_. Indeed, between 2 and 4 min, we observed a 26% increase in the lesion detection for 3D-OSEM. Second, small cancer lesions not evidenced on the CT scan alone because of their size can be evidenced on PET images if their SUVmax exceeded that measured in the vascular background. As previously reported [11], PSF reconstruction is expected to affect quantitative values in nodes < 10 mm more than 3D-OSEM. We used the vascular background because neck PRD often occurs in lymph nodes that are close to the great vessels, such as in the lateral compartment. Interestingly, our results showed that detectability increased when SUVmax values of very small lesions (i.e. not identifiable on CT scans) were above the SUVmax measured in the background which may explain the increase of lesion detection with PSF.

We used one technical advantage of our PET device, which is an extended field-of-view, enabling to increase the detection sensitivity and explore the HN region with one bed position only. Then, time acquisition was reduced compared to PET devices which require two bed positions to image the same volume. Furthermore, the small-voxel matrix of our dedicated HN PET improves lymph node detection, as previously shown in the neck [1] and in the axillary region for breast cancer [12].

This study has some limitations. First, as previously pointed out [11], PSF and OSEM images can be identified as the latter appear smoother. This could be a potential bias in the case of a more sensitive interpretation of PSF images by the reader, as reported in other clinical studies [11]. Second, the study group involved a limited number of patients, as radioiodine-refractory recurrent DTC/PDTC is a rare condition. Third, 58% of the patients benefited from pathological confirmation, but careful follow-up in the remaining 42% of patients made us confident with the ^18^FDG malignancy status. Finally, the impact of time-of-flight (TOF) was not explored. Based on a phantom study, Rogasch et al. demonstrated higher spatial resolution for PSF + TOF than PSF alone [13] but the scan time was 3 min per bed position vs. 2 to 10 in our study. It is likely that for such dedicated acquisitions with scan times of ≥ 4 min, as in our study, the lack of TOF has little impact.

## Conclusions

PSF_allpass_ HN PET improves lesion detectability as compared with 3D-OSEM HN PET. PSF_allpass_ with an acquisition time between 8 and 10 min provides the best performance in the detection of tumour recurrence.
